# Effect of the alumina micro-particle sizes on the thermal conductivity and dynamic mechanical property of epoxy resin

**DOI:** 10.1371/journal.pone.0292878

**Published:** 2023-10-13

**Authors:** Zhe Xu, Cheng Zhang, Yang Li, Jun Zou, Yuefeng Li, Bobo Yang, Rongrong Hu, Qi Qian

**Affiliations:** 1 School of Materials Science and Engineering, Shanghai Institute of Technology, Fengxian District, Shanghai, China; 2 School of Science, Shanghai Institute of Technology, Fengxian District, Shanghai, China; 3 Zhejiang Silanex Technology (Taizhou) Co., Ltd, Luqiao District, Taizhou City, Zhejiang, China; Cardiff Metropolitan University, UNITED KINGDOM

## Abstract

Epoxy thermal conductive adhesives with high thermal conductivity and dynamic mechanical properties are important thermally conductive materials for fabricating highly integrated electronic devices. In this paper, micro-Al_2_O_3_ is used as a thermally conductive filler for the epoxy resin composite and investigated the effect of micron-sized alumina particle size on the thermal conductivity and dynamic mechanical property of epoxy resin by the transient planar hot plate method and DMA (Dynamic mechanical analysis). The experimental results show that with the same amount of alumina filling, the thermal conductivity and Tg (glass transition temperature) of epoxy/Al_2_O_3_ composite material decrease with the increase of alumina particle size. The maximum thermal conductivity of the composite material is 0.679 (W/mK), while the energy storage modulus of epoxy/Al_2_O_3_ composite material increases with the increase of alumina particle size, and the maximum energy storage modulus of the composite material is 160MPa. Compared with pure epoxy resin, the thermal conductivity and energy storage modulus have increased by 2.7 and 3.2 times, respectively. The epoxy/Al_2_O_3_ composite was applied to the COB (Chips On Board) type LED package, and the substrate temperature of the LED dropped to the lowest after 1.5 hours of operation using EP-A5 composite, and the temperature was stabilized at 38.2°C, indicating that the addition of 5-micron alumina composite has the best heat dissipation in the COB type LED package. These results are critical for the implementation of particulate-filled polymer composites in practical applications because relaxed material specifications and handling procedures can be incorporated in production environments to improve efficiency.

## Introduction

Due to the rapid development of microelectronics technology, the integration [1] and miniaturized [2] of electronic components are becoming more and more advanced. This also leds to a significant increase in the heat generated by electronic devices during operation [3, 4]. Thermally conductive materials are key to heat transfer and vibration prevention of electronic components [5, 6]. Thermally conductive materials can be classified as thermally conductive pastes, thermally conductive gaskets, thermally conductive adhesives, and thermally conductive phase change materials [7]. Thermally conductive adhesives are widely used in electronic devices because of their easy processing and moulding, simple process and low cost [8]. Epoxy resins are widely used as matrix materials for thermal conductive adhesives because of their excellent mechanical properties [9], thermal stability [10], ease of preparation [11], and low cost [12]. Since the thermal conductivity of pure epoxy resin is extremely low, approximately 0.1–0.2 W/(mK) [13], the thermal conductivity of thermal conductivity adhesives prepared by filling epoxy resin with ceramic particles [14–17], carbon materials [18, 19], metal particles [20, 21] and other high thermal conductivity fillers has a significant effect, and this approach is also considered an important direction for improving the thermal conductivity of epoxy resin adhesives [22].

However, the incorporation of metal particles usually deteriorates composites’ electrical insulation and dielectric properties, limiting their wide application in electronic packaging [8, 23]. In addition, although carbon materials have the advantages of high thermal conductivity and lightweight, their high cost and low electrical insulation properties limit their practical application in the industry [24]. Ceramic particles have been widely studied for their excellent thermal conductivity and mechanical properties compared to carbon materials and metal particles [25]. Al_2_O_3_ materials have much research in thermally conductive composites due to their high thermal conductivity, low cost and stable chemical properties, making alumina a promising filler in industrial applications [26, 27]. In epoxy resin composites, alumina fillers can increase the strength and elastic modulus of the material for product molding [22, 28]. Simunin et al. [28]. added alumina nanofibers to epoxy resin. The experimental results showed that the addition of 0.2%wt alumina nanofibers increased the ultimate bending strength of the composites from 41 MPa to 71 MPa. After exceeding the penetration threshold of alumina nanofibers in epoxy resin, the bending strength of the composites decreased. The elastic modulus increased from 0.643 GPa to 0.862 GPa, which implies that alumina nanofibers have significant advantages in improving the bending strength and elastic modulus of epoxy-based composites. In addition, alumina fillers can also increase the material’s thermal conductivity for product encapsulation [29]. Yu et al. [30] added b-Al_2_O_3_ to epoxy resin to prepare 0.5 wt% b-Al_2_O_3_/EP composites, 30 wt% b-Al_2_O_3_/EP composites, and 70 wt% b-Al_2_O_3_/EP composites, and the experimental results showed that the thermal conductivity of 70% filled b-Al_2_O_3_/EP composites, the thermal conductivity of 70% b-Al_2_O_3_/EP composites is 1.13 W/(mK), which is seven times higher than that of pure epoxy resin. Zhou et al. [31] added different particle sizes of micron alumina particles to silicone rubber. The experimental results showed that the composites’ thermal conductivity increased with the alumina increase. The maximum thermal conductivity of the composites was 0.9 W/(mK), Zhou et al. further compounded alumina particles of different particle sizes in different ratios, and the results showed that the mutual doping of alumina of different particle sizes could make the silicone rubber. The composite has higher thermal conductivity, and when the mass ratio of M25*μm*/M5*μm*/M0.5*μm*/M50nm is 2:5:1:1, the thermal conductivity of the composite rubber reaches 1.45 W/(mK), which is the highest thermal conductivity among all filled rubbers, and the highest thermal conductivity when using a single particle size Al_2_O_3_. Mai et al. [32] prepared two types of micro@nanostructuredAl_2_O_3_ fillers and their epoxy resin composites. The experimental results showed that nano-Al_2_O_3_ coating in micro-Al_2_O_3_ showed that the modification effectively improved the infiltration between Al_2_O_3_ and epoxy resin, reduced interface defects caused by weak bonding between Al_2_O_3_ fillers and epoxy resin, and synergistically enhanced the thermal conductivity and mechanical properties of epoxy resin composites. The thermal conductivity of micro-Al_2_O_3_/EP, micro@nano-Al_2_O_3_/EP-6:1 and micro-Al_2_O_3_/EP, micro@nano-Al_2_O_3_/EP-3:1is 0.874 W/(mK),1.001 W/(mK), and 1.072 W/(mK), respectively. Compared with micro-Al_2_O_3_/EP, the modification effect is 11.45% and 12.27%, respectively. These above experimental results all indicate that nano-and micron-level alumina can increase the thermal conductivity of composites and improve thermal conductivity.

The construction of thermal conductivity networks in epoxy resins using nano alumina fillers with high specific surface area provides a very promising opportunity. However, nano fillers are difficult to disperse in epoxy resin and may exhibit agglomeration during the preparation process [33, 34]. It is necessary to modify the epoxy resin or nano alumina fillers, as the viscosity of nanoparticles is high, and the cost is high. Due to Micron alumina’s simple manufacturing process, the need for production equipment and technology is relatively mature, so the production cost of micron alumina is relatively low, and for the enterprise, the purchase price is low [23, 35–38]. On the other hand, due to the maturity of micron alumina production technology, micron alumina has relatively low energy consumption and emissions in the production process, reducing the waste of resources and environmental pollution in enterprises [38, 39]. Therefore, using cheap and well-flowable micron alumina and a loose processing program is a good choice. In the present study, there is a lack of research on the effect of micron alumina particle size on the performance of epoxy resin thermally conductive adhesives. Based on the above considerations, this paper focuses on preparing epoxy/Al_2_O_3_ composites with different particle sizes of micron alumina particles. It investigates the effects of different alumina particle sizes on the thermal conductivity and thermomechanical properties of epoxy/Al_2_O_3_ composites. This paper has practical significance for implementing particle-filled polymer composites in practical applications, as loose material specifications and processing procedures can be incorporated into production environments to improve efficiency.

## Materials and methods

### Materials

The epoxy resin is bisphenol A epoxy resin (NPEL-128E type) produced by Nanya Electronic Materials Co., Ltd. The curing agent was 2-Aminoimidazole (GY HT110 type) manufactured by Guangzhou Guyan Electronic Materials Co., Ltd. The diluent is 4-tert-butylphenyl glycidyl ether (type 692) produced by Suzhou Senfida Chemical Co., Ltd. The thermally conductive filler was Al_2_O_3_ with 99.9% purity and particle sizes of 5,10,40,60, and 120 *μ*m from Sumitomo Chemical Co., Ltd. The surface modifier was pyrolytic silica (HDK H18 type) from Wacker Chemicals (Cinha) Co., Ltd.

### Preparation of micro-composite samples

**Fig 1:**
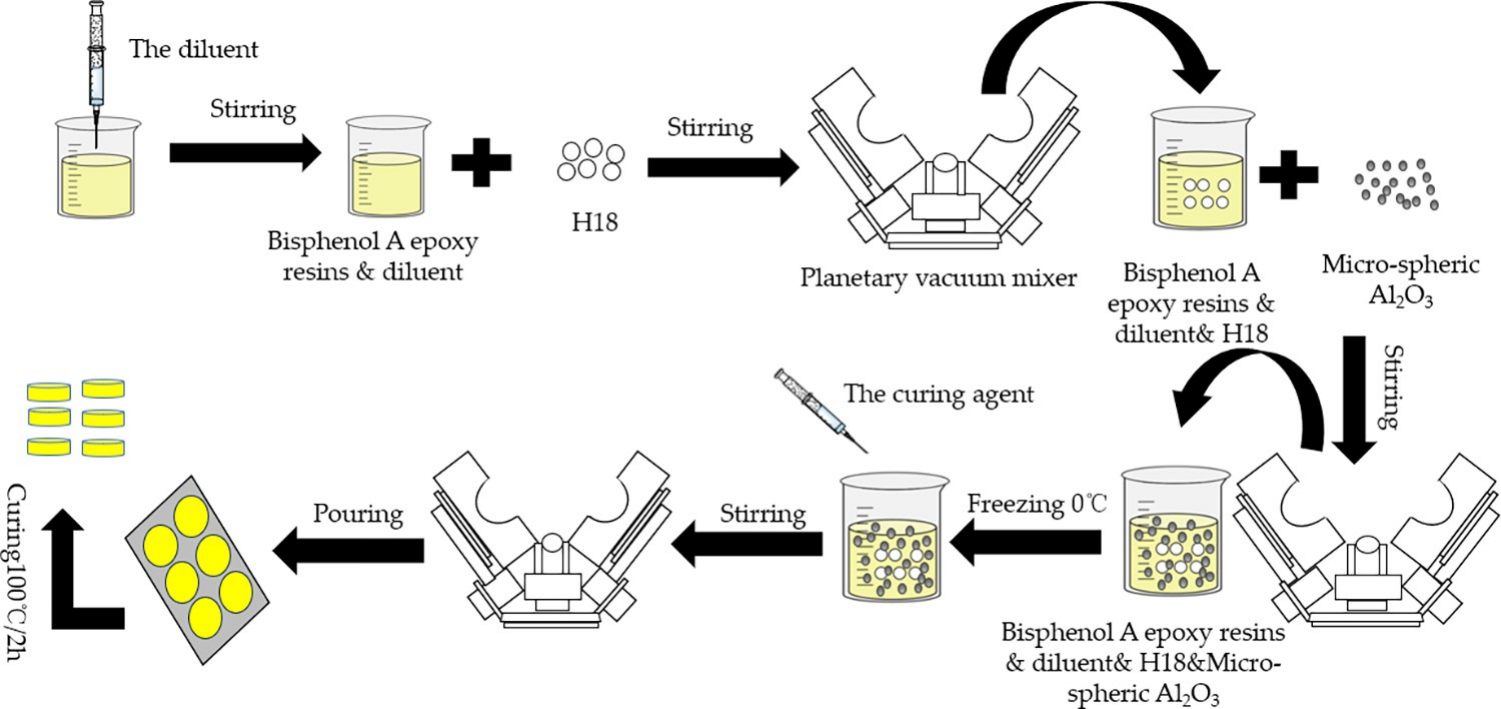
Flow chart of sample preparation.

**Fig 2:**
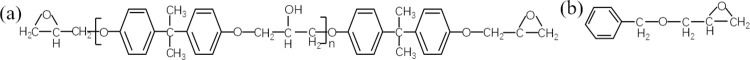
The chemical structure of the epoxy resin (a) and (b) diluent.

Preparation of epoxy resin composites filled with different particle sizes of alumina using a planetary stirring device (Zhongmix Technology Co., Ltd., Mianyang, Sichuan, China). First, 41.5vol.% liquid bisphenol A epoxy resin and 5 vol.% diluent were added to the beaker and stirred for 5 minutes. Then add 1 vol.% H18 and blitz with a planetary blender for 10 minutes. Next, 50 vol.% alumina was added and placed in the planetary stirring device for 10 minutes, vacuuming for 10 minutes. The mixture was cooled to 0°C in a refrigerator, and finally, the curing agent was added and stirred and mixed for 15 minutes, molded on glass and cured at 100°C for 2h to prepare the cured composite. The flow chart of the sample preparation was shown in Fig 1. Table 1 Sample numbers for different alumina particle sizes. The chemical structure of the epoxy resin and diluent is shown in Fig 2.

**Table 1:** Sample names for different alumina particle sizes.

The name of samples	Al_2_O_3_ particle sizes(*μ*m)	Al_2_O_3_ volume
EP	0	0 vol.%
EP-A5	5	50 vol.%
EP-A10	10	50 vol.%
EP-A40	40	50 vol.%
EP-A60	60	50 vol.%
EP-A120	120	50 vol.%

#### FTIR spectrometer tests

Infrared spectra were obtained from a Nicolet 380 FTIR spectrometer (Nicolet Instrument, Madison, American) with an accumulation of 32 scans at a resolution of 0.5cm^-1^. Samples were mixed with KBr powder and pressed into a disc.

### Electron microscopy

The microscopic morphology of the composite cross-section was studied using a scanning electron microscope (FEI Quanta200 FEG, Eindhoven, The Netherlands).

### Thermal conductivity tests

Thermal conductivity was measured using a thermal conductivity meter (Hot Disk TPS 2500S, Uppsala, Sweden) at room temperature using the transient planar hot plate method in the direction perpendicular to the sample plane.

### Dynamic mechanical analysis

The dynamic mechanical property of epoxy/Al_2_O_3_ micro-composites was tested using the DMA242C instrument from NETZSCH. The mechanical modulus was obtained by simultaneous testing of stress and strain. The temperature range was 40°C-200°C, the heating rate was 5 K/min, the frequency was 1 Hz, the measured sample size was 10 x 10 x 2 (thickness) mm^3^, the applied force was 5 N, and the amplitude was 40 *μ*m.

### Temperature recording

The epoxy/Al_2_O_3_ composite material is bonded between the COB-type substrate and the luminaire housing, and the temperature of the selected points of the substrate and housing is tested using a TP1000 temperature data logger produced by Shenzhen Topley Electronics Co. The driving voltage of COB is 50V, the current is 1.2A, the power of COB type LED is 60W, and the working time is 1.5h.

## Results and discussion

### FTIR and EDS mapping analysis

**Fig 3:**
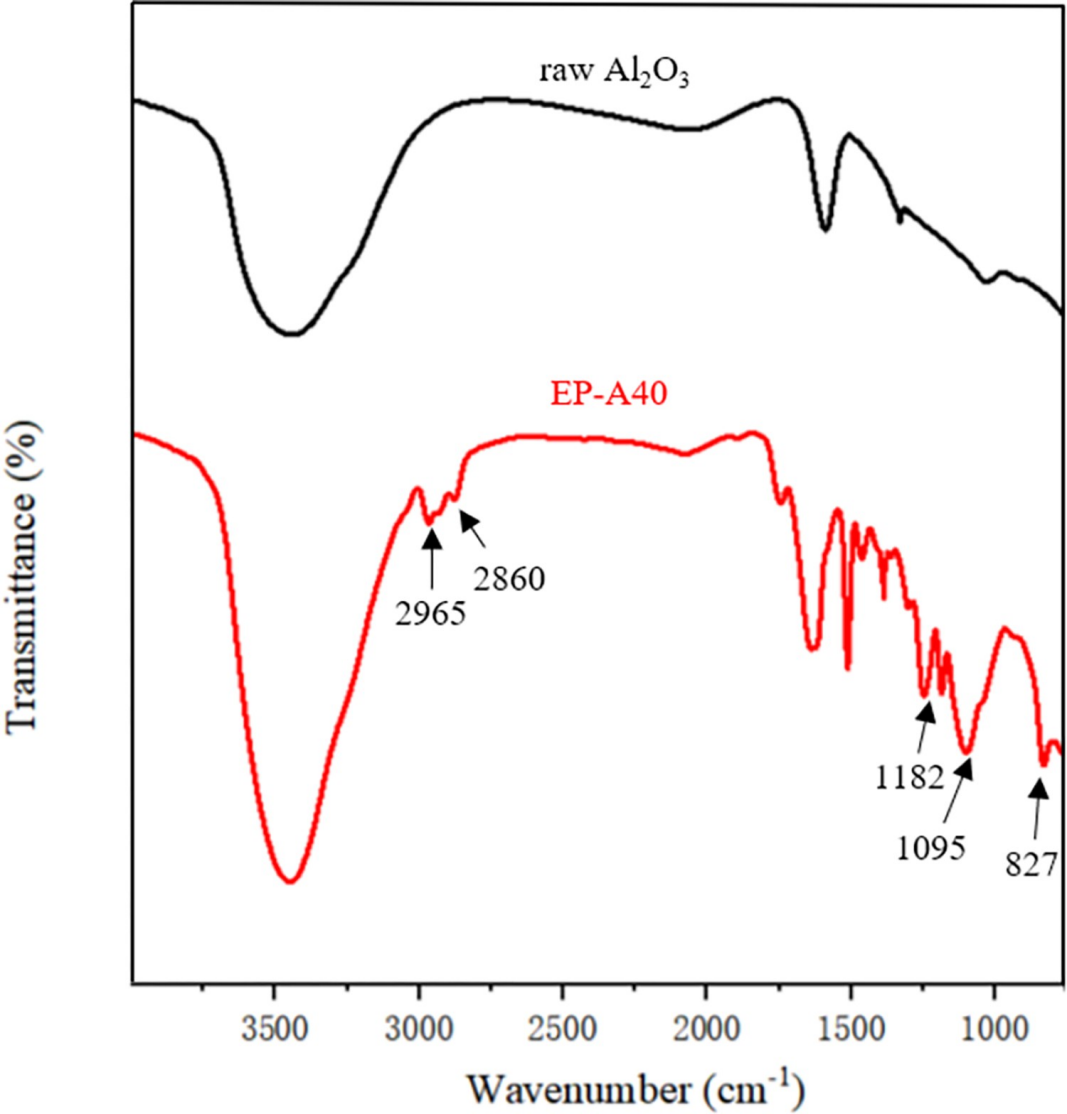
FTIR spectra of raw Al_2_O_3_ and EP-A40.

**Fig 4:**
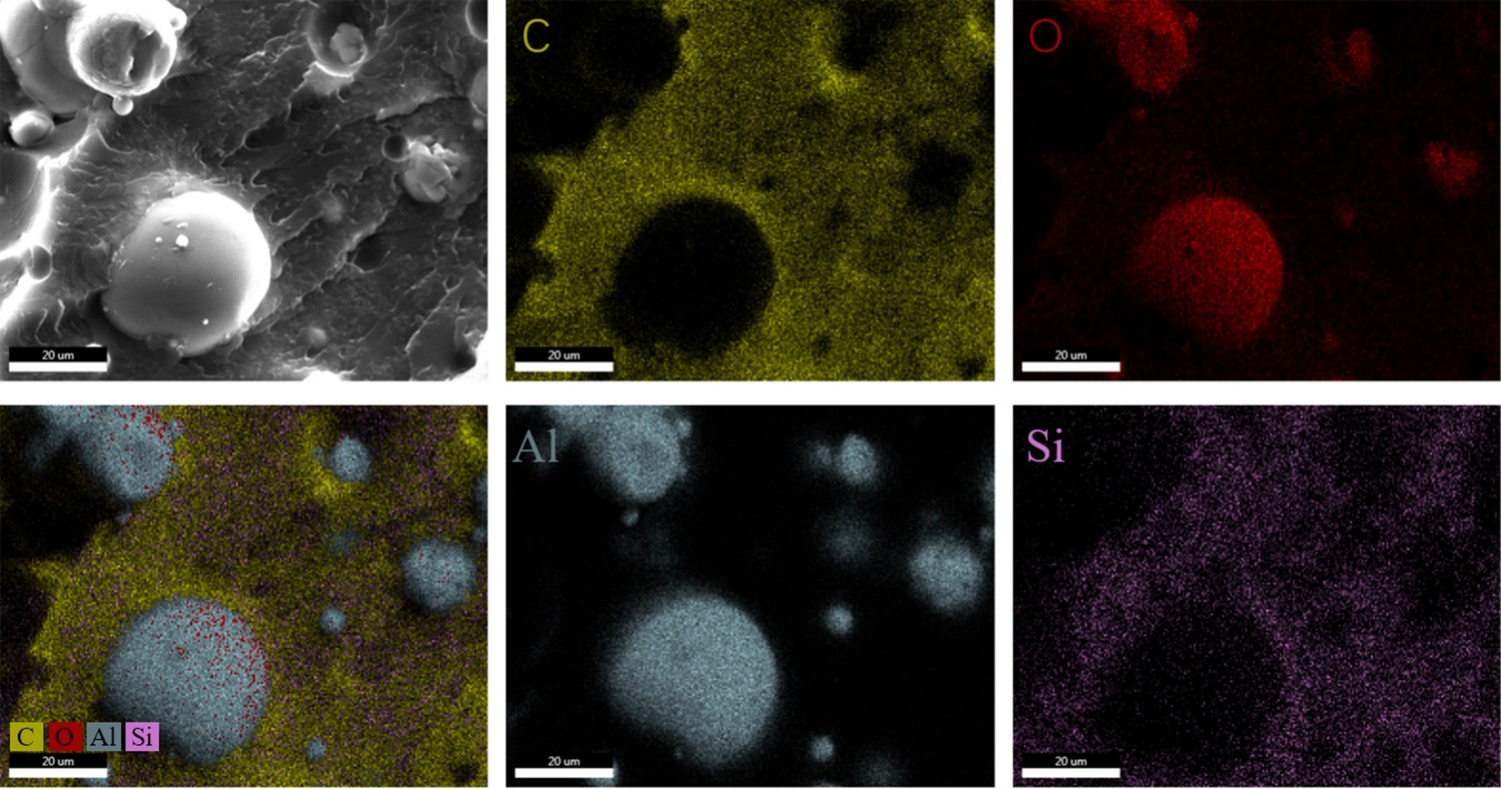
SEM images of EP-A40 and its corresponding EDS elemental mappings of C, O, Al and Si.

**Fig 5:**
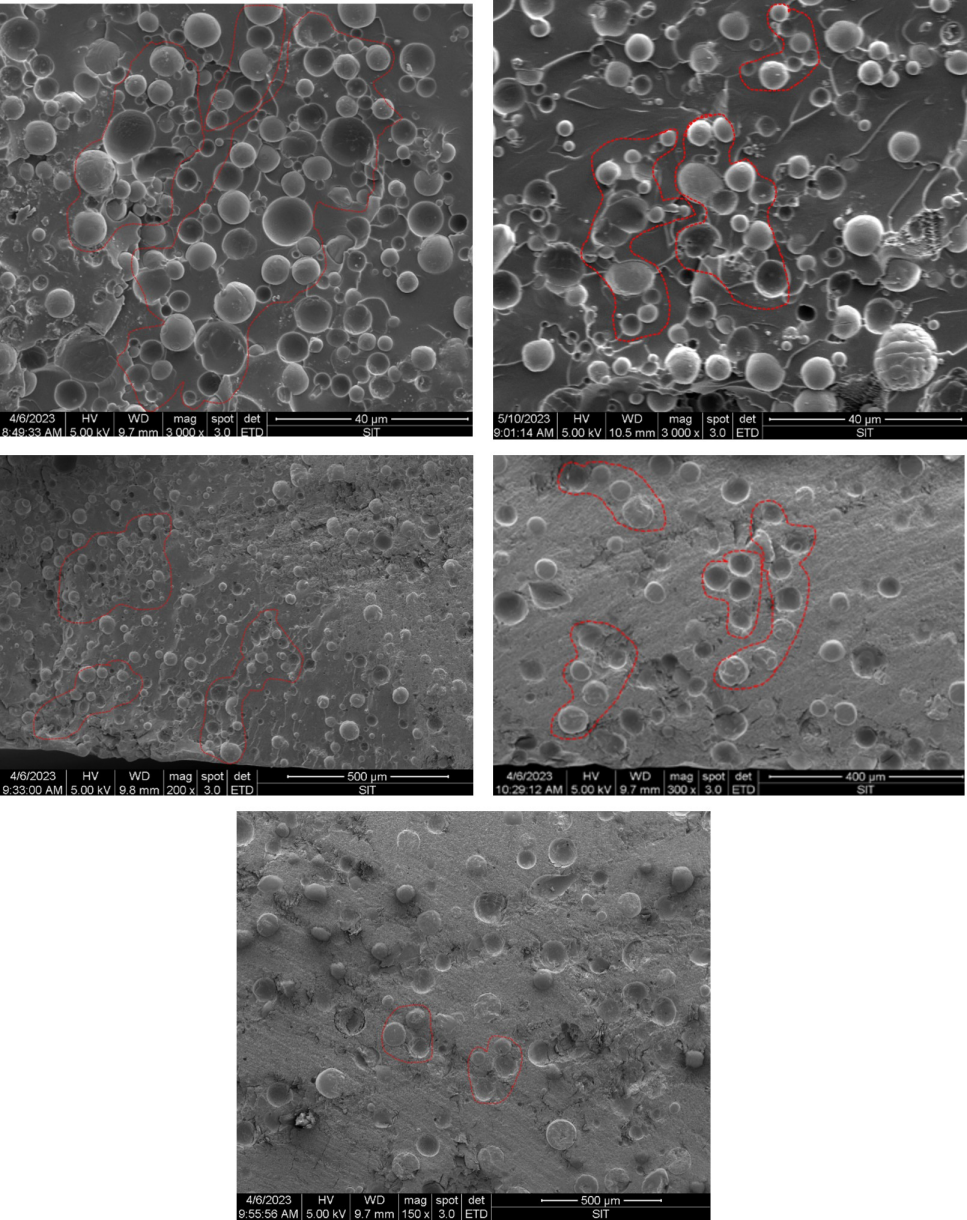
Epoxy resin micron composites morphology: (a)EP-A5; (b)EP-A10; (c)EP-A40; (d)EP-A60; (e)EP-A120. The red area indicates a part of the stacked structure.

To demonstrate the actual occurrence of chemical reactions between epoxy resin and Al_2_O_3_, FTIR spectra of functionalized Al_2_O_3_ were recorded in Fig 3. Compared with pristine Al_2_O_3_, EP-A40 exhibits new absorption at 2965 and 2860 cm^-1^, which represent the valence stretching vibration of aliphatic C-H. The peaks at 1182,1095 and 827 cm^-1^ are assigned to the C-O-C stretch, Si-O-Si stretch, and epoxy group, respectively. The result of FTIR indicate that the epoxy group has been successfully introduced into the surface of Al_2_O_3_ particles through chemical grafting [26, 27, 40]. The obtained sample was characterized by SEM. Fig 4 shows the SEM image of the EP-A40 composite along with the corresponding EDS elemental mappings of aluminum (Al), oxygen (O), carbon (C) and silicon (Si). Figs 4 and 5C further illustrates that the Al_2_O_3_ granules pack together more continuously and closely to fill the space of epoxy resin structure.

### Thermal conductivity analysis

The thermal conductivity of Al_2_O_3_ (30–40 W/mK) is much larger than that of pure epoxy resin (0.1–0.2 W/mK). Therefore, the addition of Al_2_O_3_ to the epoxy resin matrix will improve the thermal conductivity of the epoxy resin, and the particle size of Al_2_O_3_ will affect the thermal conductivity of the composite epoxy resin.

The dispersion of alumina particles varies with particle size, which affects the thermal conductivity of the composites. When the alumina addition is the same, the dispersion of alumina particles varies with the particle size. Fig 5A–5E shows SEM micrographs of the composites with particle sizes of 5, 10, 40, 60 and 120*μm* alumina particles filled with epoxy resin, respectively. The average interparticle distance decreases with decreasing particle size, and smaller particle size provides more linked alumina particles. Most of the alumina particles in EP-A120 are uniformly dispersed in the matrix without agglomeration, and the particles are not interconnected (shown in Fig 5E), while more alumina particles connected with the same content can be found in EP-A40. In the EP-A5 composite, the 5 *μm* alumina particles overlapped and interwoven in the matrix, forming a dense and continuous stacking structure, which facilitates an effective heat transfer channel within the material.

**Fig 6:**
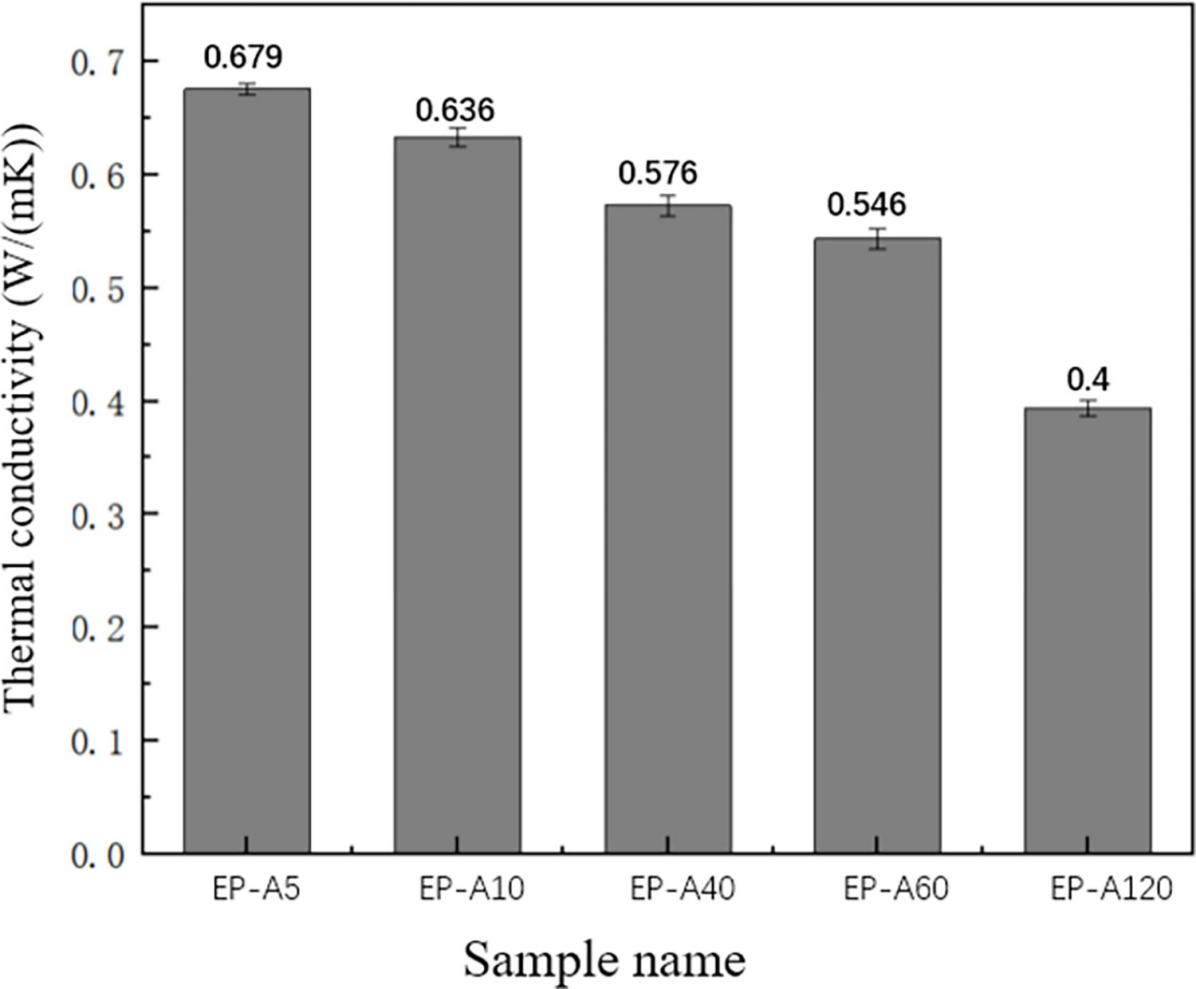
Effect of different particle sizes of Al_2_O_3_ on the thermal conductivity of epoxy resin.

**Fig 7:**
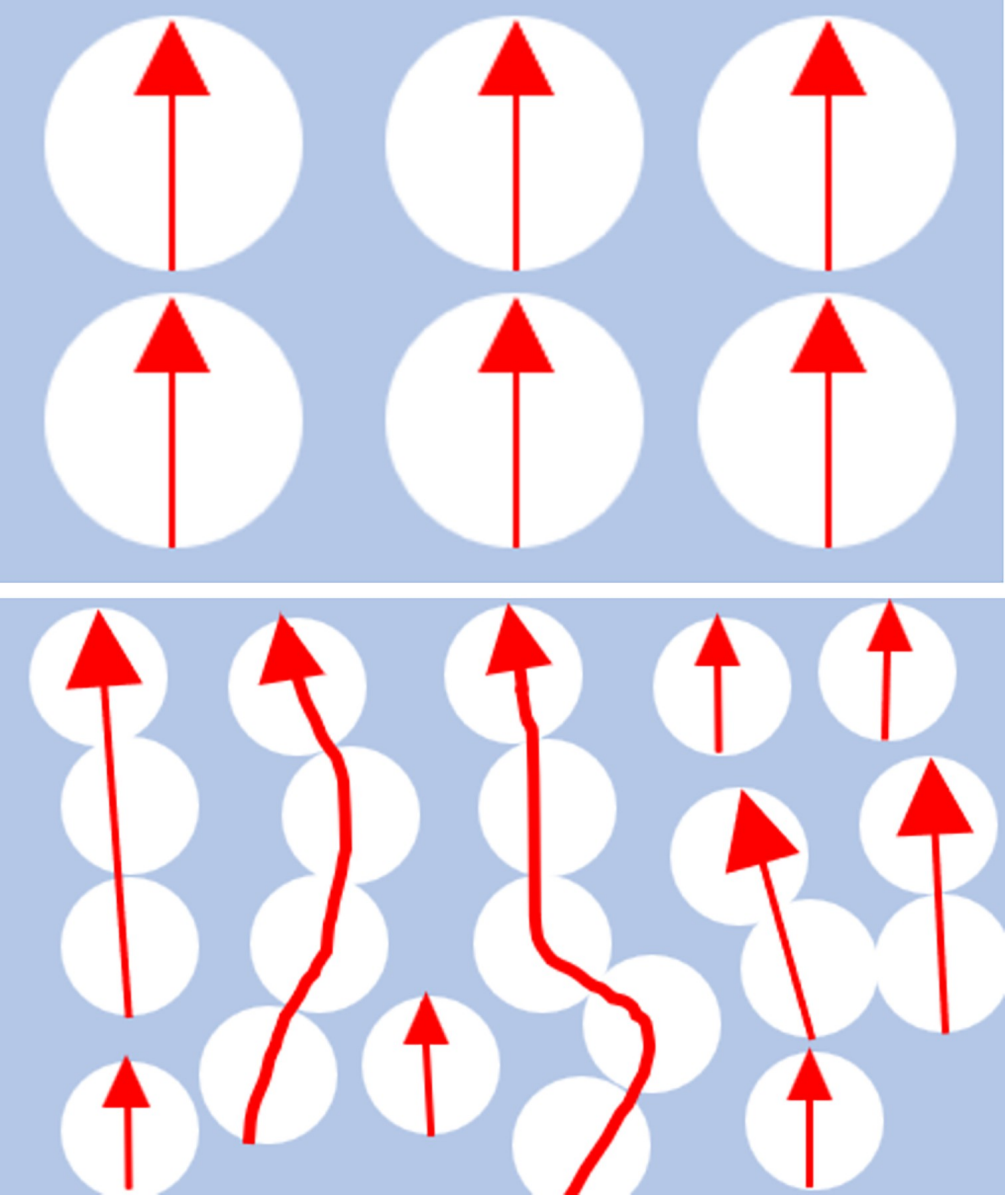
Heat transfer model of filled composites: (a) large particle; (b) small particle. Red arrows are heat transfer paths.

As can be seen from Fig 6, EP-A5 has the highest thermal conductivity of 0.679 W/(mK), EP-A10, EP-A40 and EP-A60 have thermal conductivities of 0.636 W/(mK), 0.576 W/(mK) and 0.546 W/(mK), respectively. In contrast, EP-A120 has a much lower thermal conductivity than the other four groups of composites, with thermal conductivity of 0.4 W/(mK). Among them, the thermal conductivity of EP-A5 and EP-A10 did not differ much. These results show a relationship between the thermal conductivity of alumina and its particle sizes, with smaller particle sizes having better thermal conductivity. This is because alumina with a smaller particle size has a higher specific surface area and can transfer heat more efficiently to the surrounding environment. A temperature gradient was created at the surface of the alumina particles when heat input was applied. This causes heat to be transferred from the surface to the particle’s interior. In the case of smaller alumina particle sizes, heat can be transferred more rapidly to the center of the particle and then to the surroundings via interparticle contact. Heat transfer is slower for larger alumina particle sizes because heat must travel a greater distance to reach the center of the particle. For epoxy/Al_2_O_3_ composite materials, the thermal conductivity of epoxy resin filled with large-size alumina particles is always lower than that of epoxy resin filled with small-size alumina particles [31, 41]. This is because at the same alumina filling concentration, the particles with the worst thermal conductivity (large-size alumina particles) cannot form a dense stacking structure, and some of them are even completely wrapped by the epoxy resin matrix (as shown in Fig 5D and 5E). Different particle sizes in the same epoxy matrix fraction will result in different interfacial areas per unit volume. As the particle size increases, the interfacial area between the particles and the resin matrix also increases; with the same volume fraction, a thicker epoxy resin layer may exist around the larger particles, and a thinner epoxy resin layer around the smaller particles, and the small particles stack on top of each other in contact with each other, as shown in Fig 7A and 7B. Therefore, the smaller particles are easier to form the thermal conductivity channel, which is more stable. The experimental results show that filling with small alumina particles is easy to start a dense stacking structure, and these dense stacking structures constitute a stable thermal conductivity network [31]. The smaller the particles are, the better the thermal conductivity of the composites, and filling smaller particles is an effective way to improve the thermal conductivity of the composites.

### Thermal conductivity of composite materials in COB-type LEDs

Due to their high efficiency and compact size, LEDs are predicted to be the next generation of lighting electronics, and they are becoming a demand in daily social life and can be seen everywhere in society [42]. COB-type LEDs (COB (Chip on Board) LED lamps) with high brightness, high color temperature, and high CRI are widely used in various fields, such as indoor lighting, automotive lighting, and stage lighting [43]. However, COB-type LEDs still have thermal problems in high-power applications. Thermally conductive adhesives and tin-based solders are widely used to bond the LED chips to the substrate and to dissipate the heat generated by the LED chips through the substrate.

**Fig 8:**
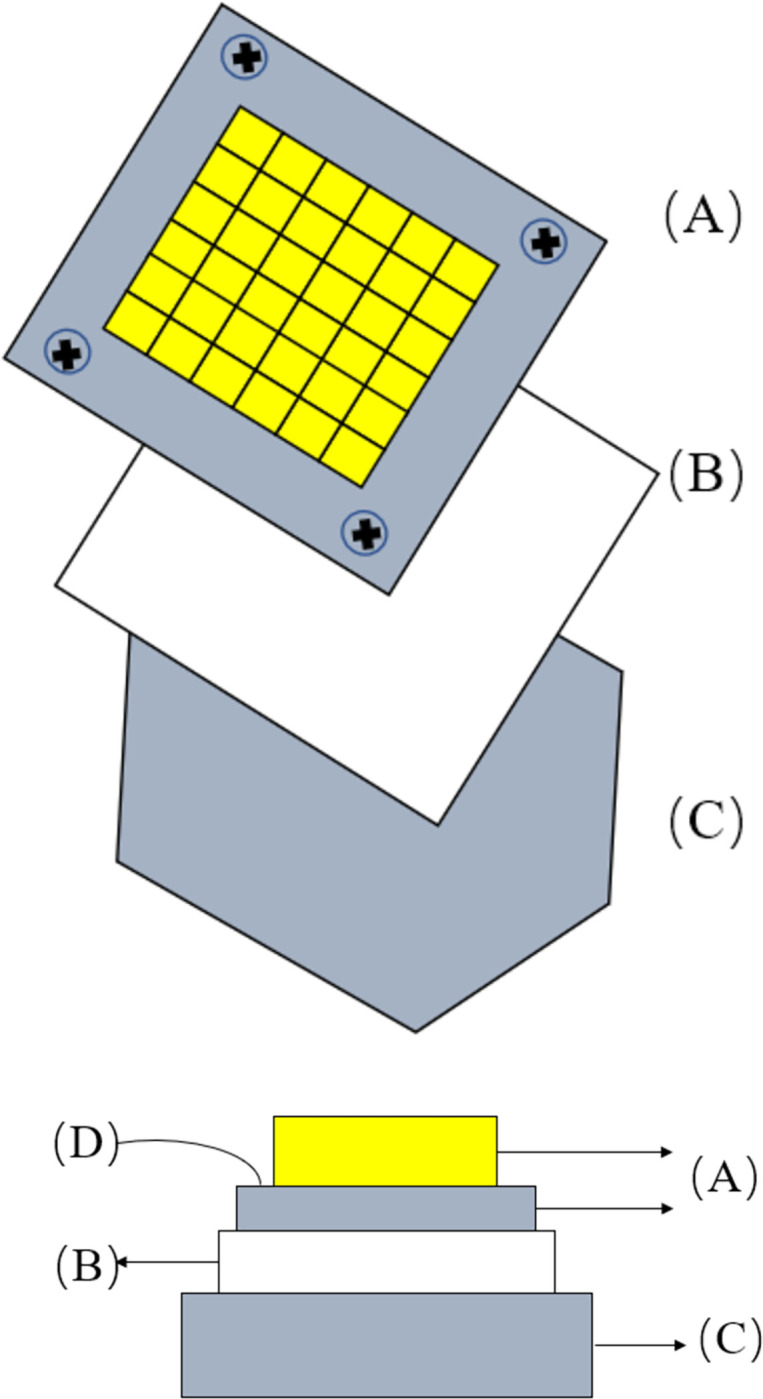
Sample placement diagram: (a)structure view, (b) side view; where (A) COB package board, (B) sample thermal adhesive, (C) housing, (D) temperature measurement point.

**Fig 9:**
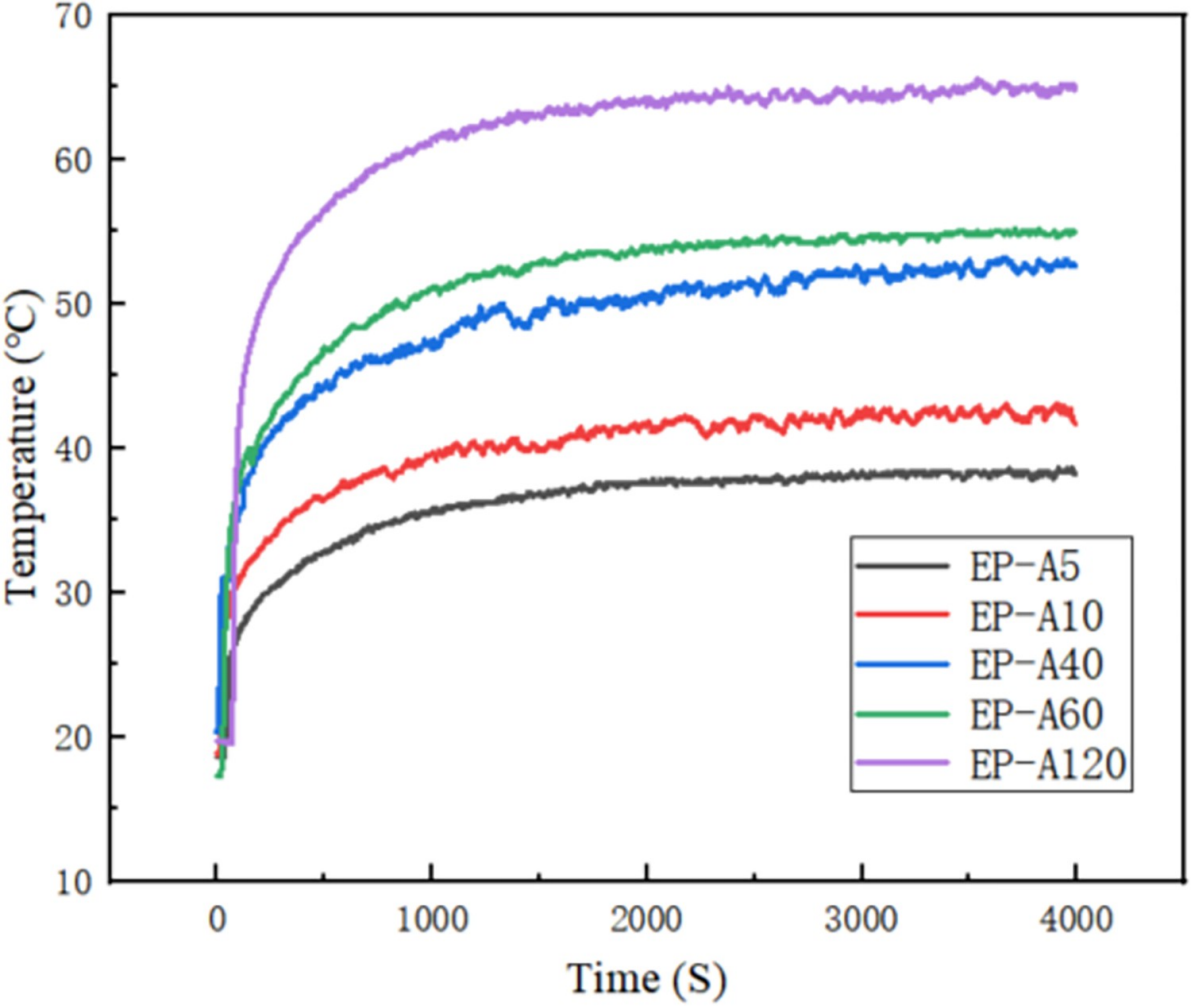
Temperature variation of LED substrate using different samples of thermally conductive adhesives.

**Fig 10:**
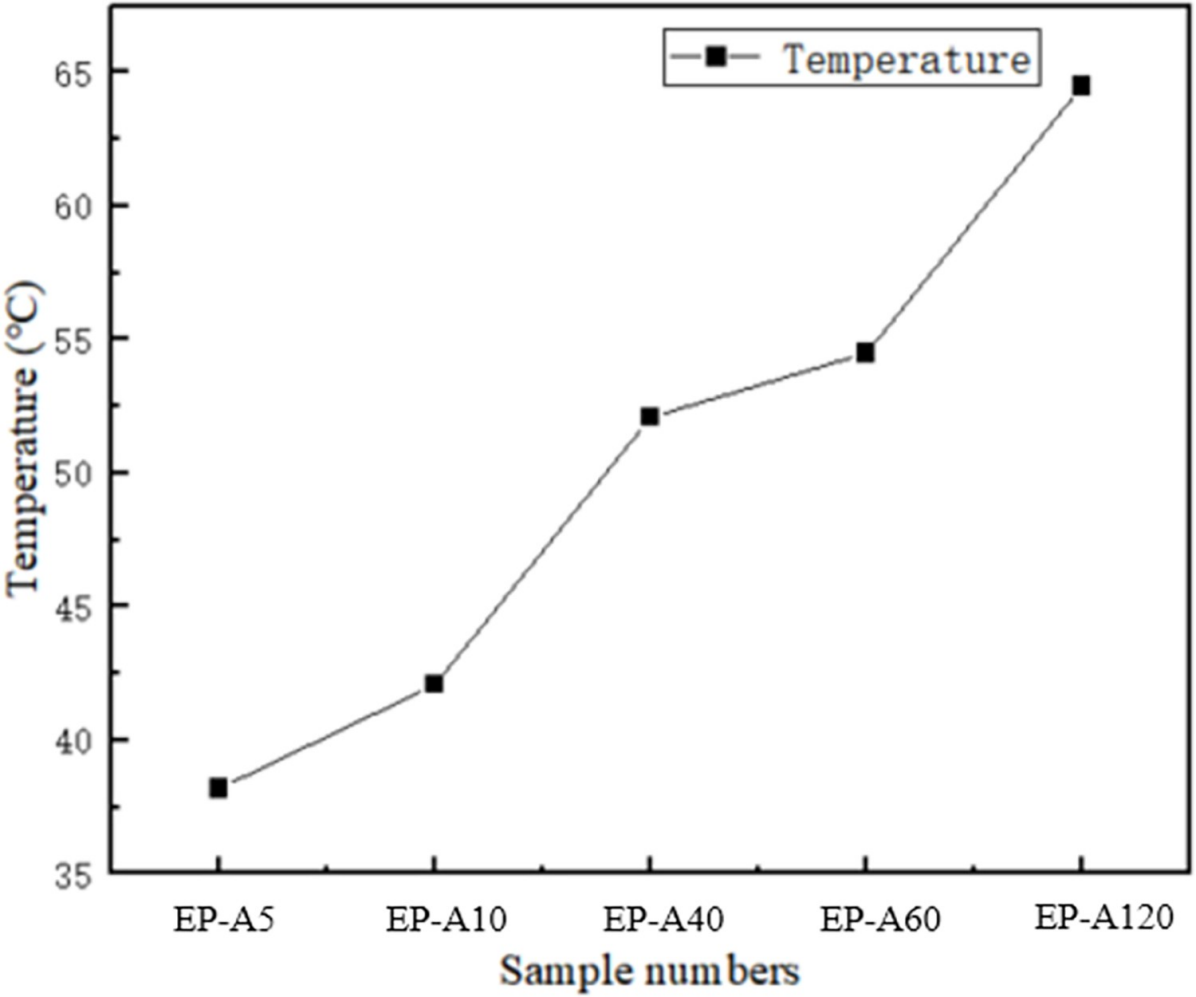
COB substrate stable temperature with different particle sizes of thermally conductive adhesive.

As shown in Fig 8, the LED COB package board is bonded to the housing as a whole through a thermally conductive adhesive. The heat from the COB substrate is transferred to the accommodation through the thermally conductive adhesive, and the thermal conductivity of the adhesive determines the final heat dissipation temperature of the substrate. The temperature of the substrate is recorded by changing different thermally conductive adhesives (Fig 9). The temperature of the substrate increases with the increase of light time, and in the first minute of light, the temperature of the substrate rises rapidly, as shown in Fig 8. This is because LEDs generate much heat when operating, and this heat is transferred to the substrate, then to the housing through the thermal conductive adhesive, and finally to the environment. With the increase of light time, the substrate temperature gradually tends to stabilize, and after 1.5h of light, the final substrate temperature of EP-A5 is stabilized at 38.2°C, and EP-A10, EP-A40, EP-A60 and EP-A120 are stabilized at 42.1°C, 52.1°C, 54.5°C and 64.5°C, respectively, as shown in Fig 10. The substrate temperature change and final stabilization of EP-A5 temperature are much smaller than the other four groups of samples, which can keep the substrate at a lower temperature when the LED works for a long time and avoid damage caused by heat accumulation.

### Dynamic mechanical property of epoxy/Al_2_O_3_ composites

Dynamic thermo-mechanical analyzers (DMA) are widely used in the study of viscoelastic properties of materials to measure data such as dynamic energy storage modulus (E′), loss modulus (E″) and loss tangent angle of materials under vibrational loading, as well as to test the glass transition temperature Tg value of materials. The glass transition temperature (Tg) is an important parameter affecting the material’s process and service performance [44]. The DMA method investigated the effect of different particle sizes of Al_2_O_3_ on the thermomechanical properties of EP composites.

**Fig 11:**
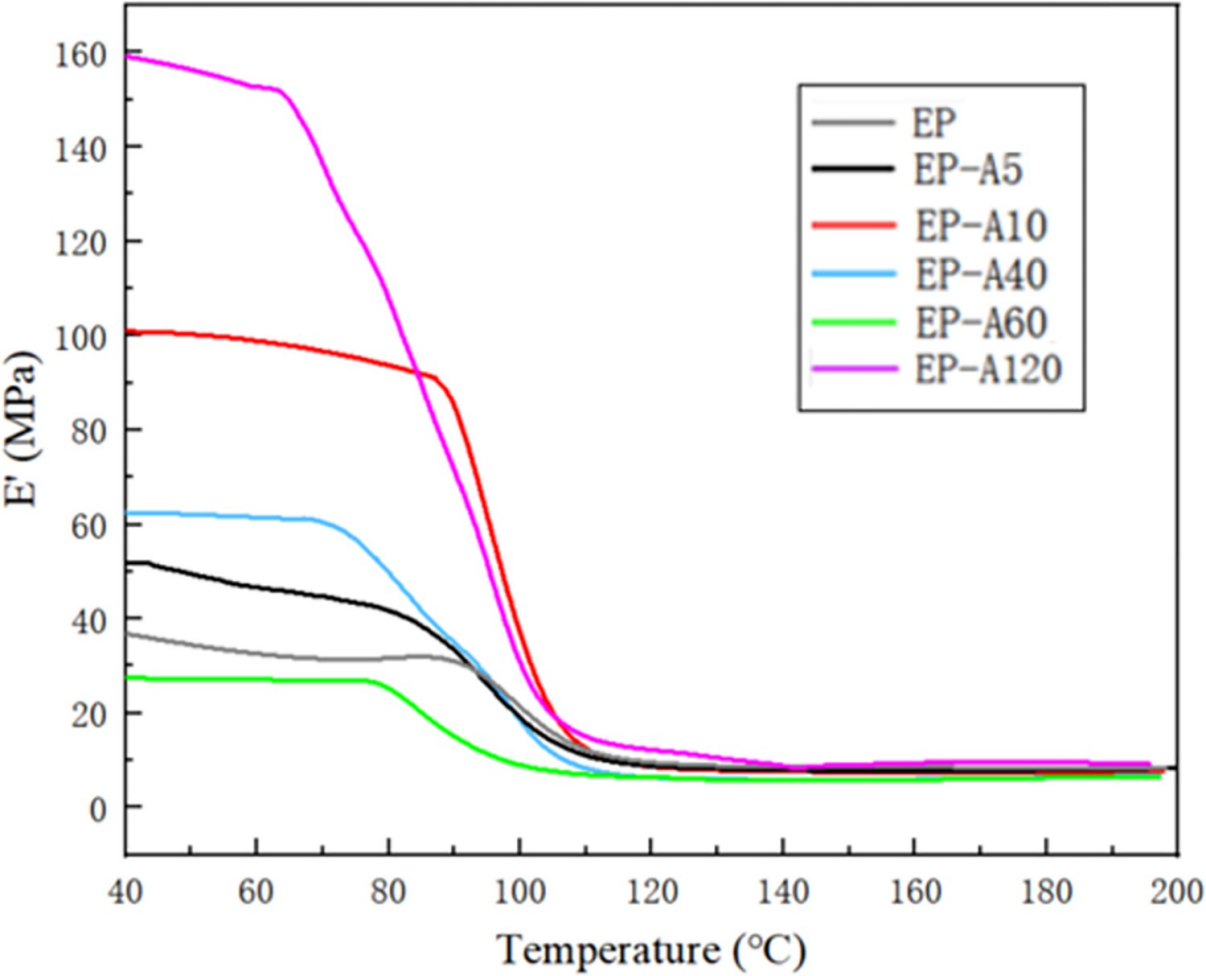
The storage modulus curve of epoxy/Al_2_O_3_ composites.

**Fig 12:**
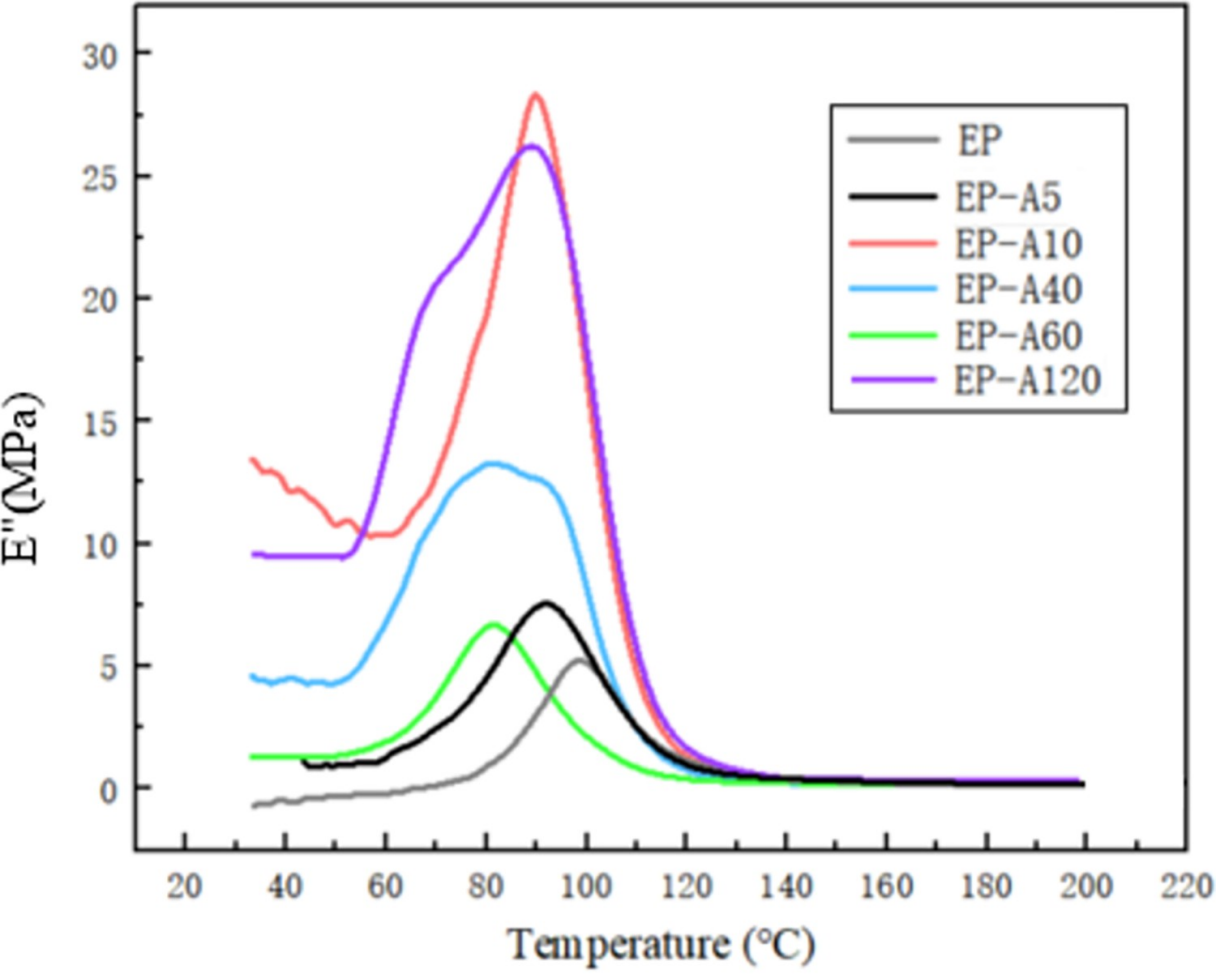
The loss modulus curve of epoxy/Al_2_O_3_ composites.

Figs 11 and 12 show the E′ and E″ curves of EP and its micro-composites. The energy storage modulus of the epoxy resin and its micro-composites decreases with increasing temperature, as shown in Fig 11. The viscoelasticity of the epoxy resin can explain this. EP and its micro-composites exhibit a glassy state with high elasticity at low temperatures, resulting in higher E′, and EP and its micro-composites exhibit high viscosity with low elasticity at high temperatures, resulting in lower E′. In the low-temperature region (<~80°C), the E′ of EP and its micro-composites slightly decreased with the increase in temperature. The addition of alumina increases E′, which increases and then decreases with increasing temperature. The energy storage modulus of EP-A5, EP-A10, EP-A40, and EP-A120 composites reach 51.8 MPa, 100.9 MPa, 62.6 MPa, and 160 MPa at 40°C, respectively (shown in Table 2), which are increased compared with pure EP by 37.4%, 167.6%, 66%, and 324.4%, respectively, compared with pure EP. At high temperatures (>~80°C), E′ decreases significantly with increasing temperature but does not change much with increasing particle size. There is a significant transition of EP between 85°C and 103°C, which indicates a thermal transformation of the molecular chain, such as a glass transition process. Fig 12 shows the variation of E″ with temperature, with a significant peak in EP near 98.5°C, corresponding to a significant decrease in E′ of EP in Fig 12. Thus the glass transition temperature of EP is 98.5°C. The glass transition temperatures of EP-A5, EP-A10, EP-A40, EP-A60 and EP-A120 are 92.4°C, 90.1°C, 82.1°C, 82.6°C and 89.1°C, respectively. The above results indicate that the incorporation of different particle sizes of alumina can change the energy storage modulus and Tg value of the epoxy resin due to the modification of the composites’ molecular chain relaxation by adding alumina. The incorporation of alumina with a particle size of 5 *μm* increases the energy storage modulus of epoxy/Al_2_O_3_ composites and has a small effect on Tg.

**Table 2:** Information on modulus and glass transition.

The name of samples	E′(MPa)at 40°C	E″ of peak temperature (°C)
EP	37.7	98.5
EP-A5	51.8	92.4
EP-A10	100.9	90.1
EP-A40	62.6	82.1
EP-A60	27.4	82.6
EP-A120	160	89.1

Figs 11 and 12 show that the curing process is divided into two stages. The first stage is 0 < T < Tg, in which the composite is in the glassy state. As the temperature increases, the energy storage modulus of the composite gradually decreases, and around Tg, the energy storage modulus starts to fall sharply while the loss modulus increases sharply. This is because, with the increase in temperature, the polymer chain segments change from frozen to mobile, and the molecular chain segments start to move. The crosslink density gradually increases, which leads to the increase of frictional resistance within the chain segment movement. Most of the energy is dissipated in exothermic heat, resulting in a sharp decrease in energy storage modulus and a sharp increase in the loss modulus of the composite material. The second stage is when Tg < T < Tf, the composite material enters a high elasticity state. With the increase in temperature, the curve tends to flatten out. This is because the crosslink density has reached a high level, and the movement of chain segments becomes difficult. The curing reaction is basically completed, and the curves of energy storage modulus and loss modulus become flat as the temperature continues to increase.

## Conclusions

This article investigates the thermal conductivity and dynamic mechanical properties of five different micrometer alumina particle-doped epoxy resin composites. The thermal conductivity results indicate that many cheap small-sized spherical alumina as the main body of the thermal conductivity network can more effectively form thermal conductivity paths, significantly improving the thermal conductivity performance of epoxy composite materials. Forming an effective thermal path is a critical factor in determining thermal conductivity. In addition, DMA results revealed molecular motion at the interface between micrometer alumina particles and EP matrix. Adding alumina particles can increase the storage modulus of epoxy/Al_2_O_3_ composite materials and reduce their Tg. Using a particle size of 5*μm* Al_2_O_3_ can effectively improve the storage modulus of epoxy resin/Al_2_O_3_ composite materials with little impact on the Tg of the composite material, which is beneficial for its application as a thermally conductive material in electronic packaging. Based on our research results, considering micron fillers may be a wise decision when designing high-performance epoxy-based electronic packaging materials with ideal comprehensive performance and balancing other functional requirements such as thermal conductivity and low cost.

## Supporting information

S1 File Original SEM images of EP-A5, EP-A10, EP-A40, EP-A60 and EP-A120 composite materials. (DOCX)

S2 File Original file: SEM images of EP-A40 and its corresponding EDS elemental mappings of C, O, Al and Si. (DOCX)
