# Supplementary material for: Effect of the alumina micro-particle sizes on the thermal conductivity and dynamic mechanical property of epoxy resin
Source: PLoS One. 2023 Oct 13;18(10):e0292878. doi: 10.1371/journal.pone.0292878 (PMC10575537; doi:10.1371/journal.pone.0292878)

Original SEM image of EP-A5 composite material (Figure 5 a)


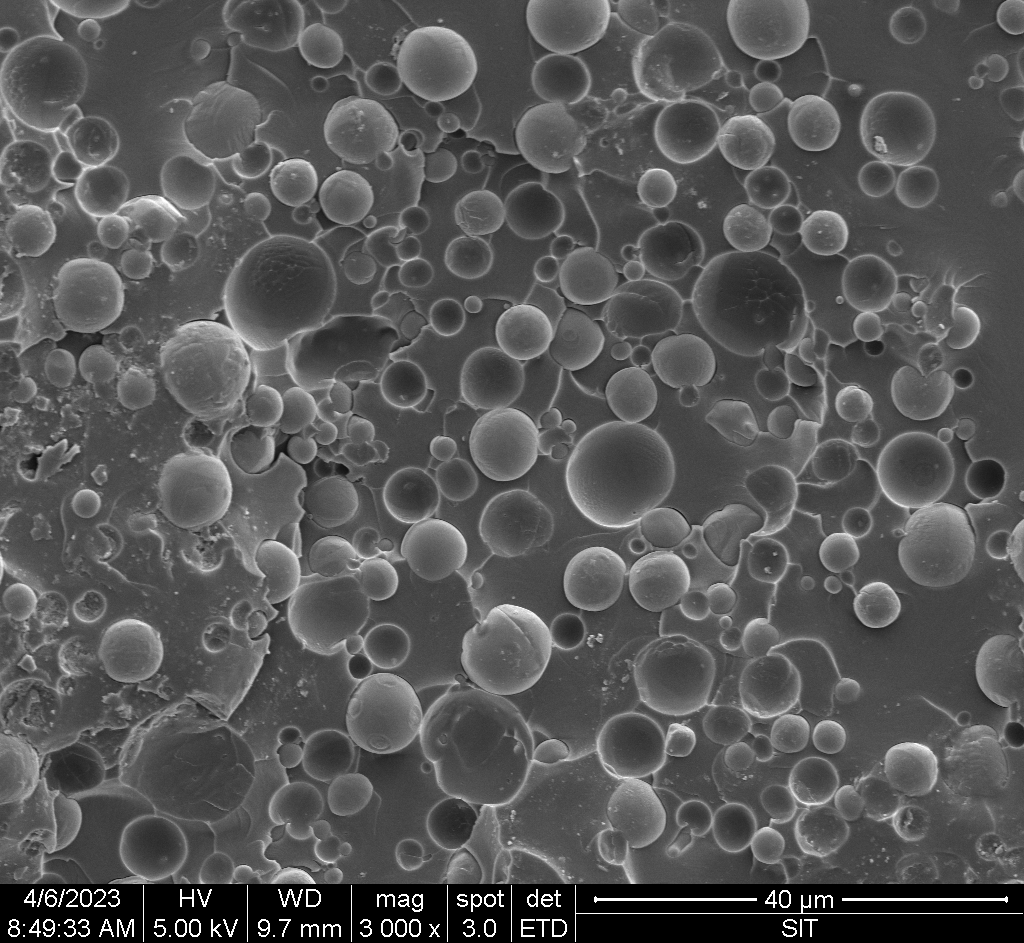


Original SEM image of EP-A10 composite material (Figure 5 b)


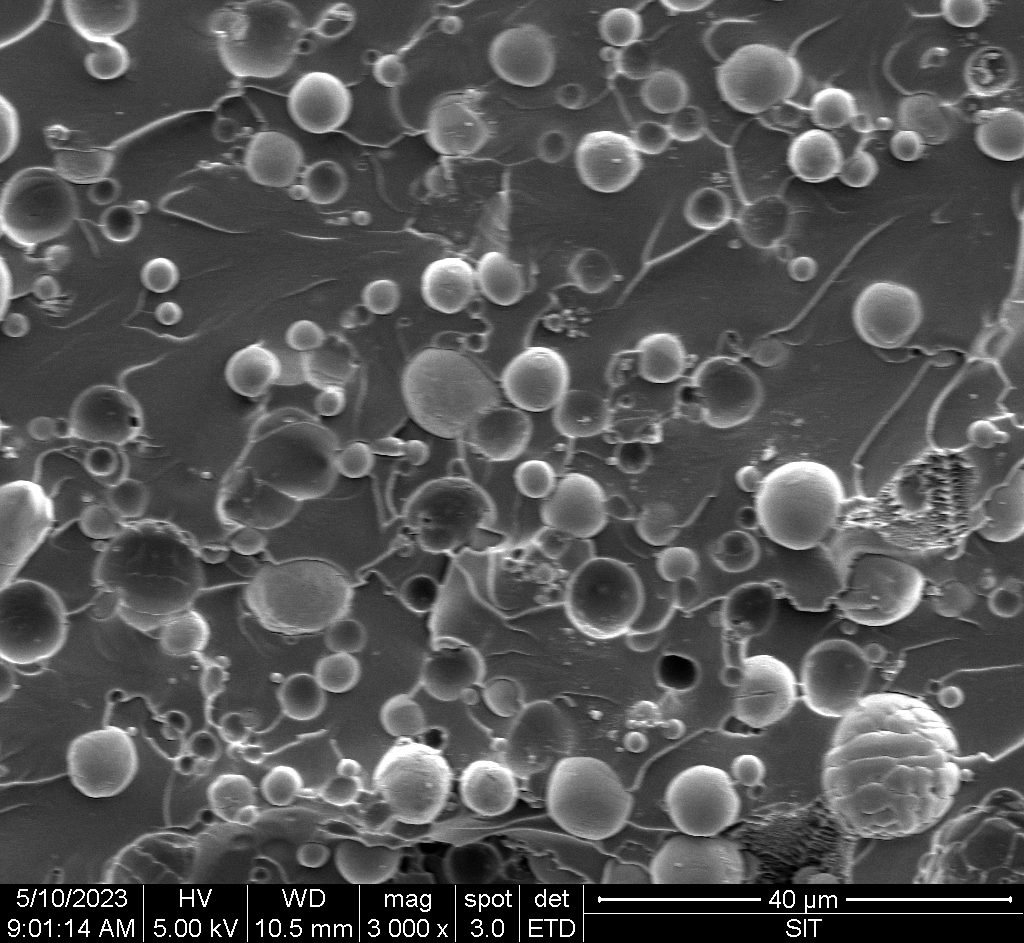


Original SEM image of EP-A40 composite material (Figure 5 c)


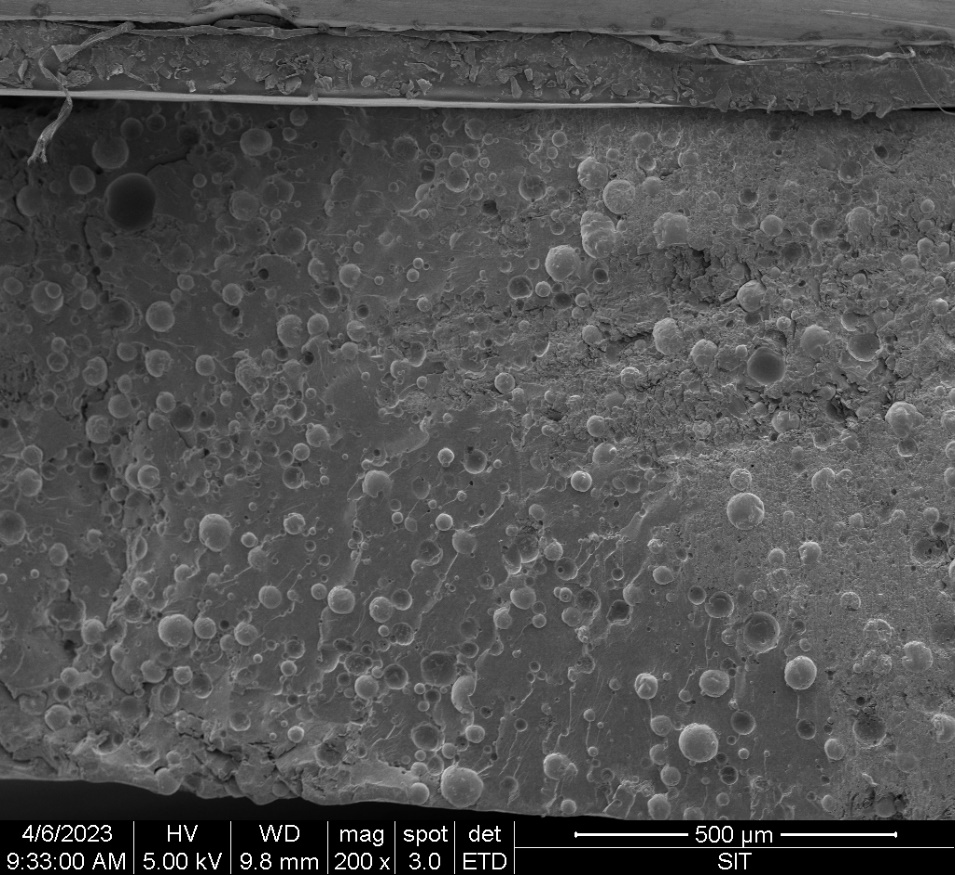


Original SEM image of EP-A60 composite material (Figure 5 d)


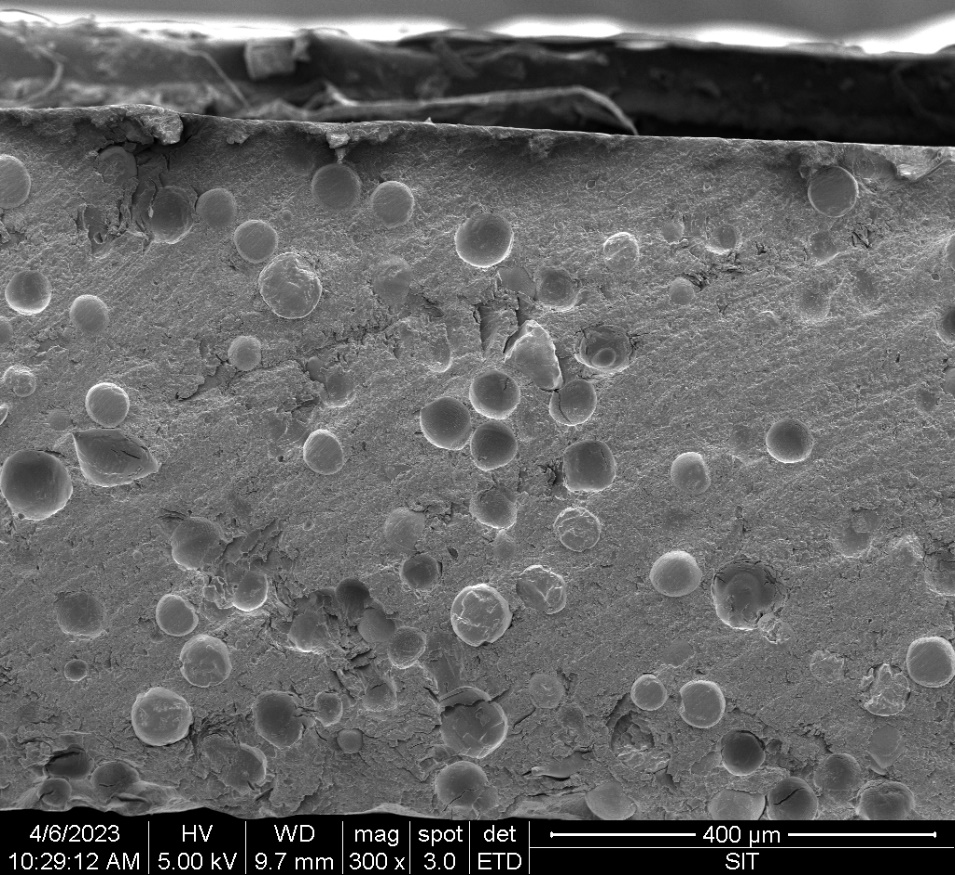


Original SEM image of EP-A120 composite material (Figure 5 e)


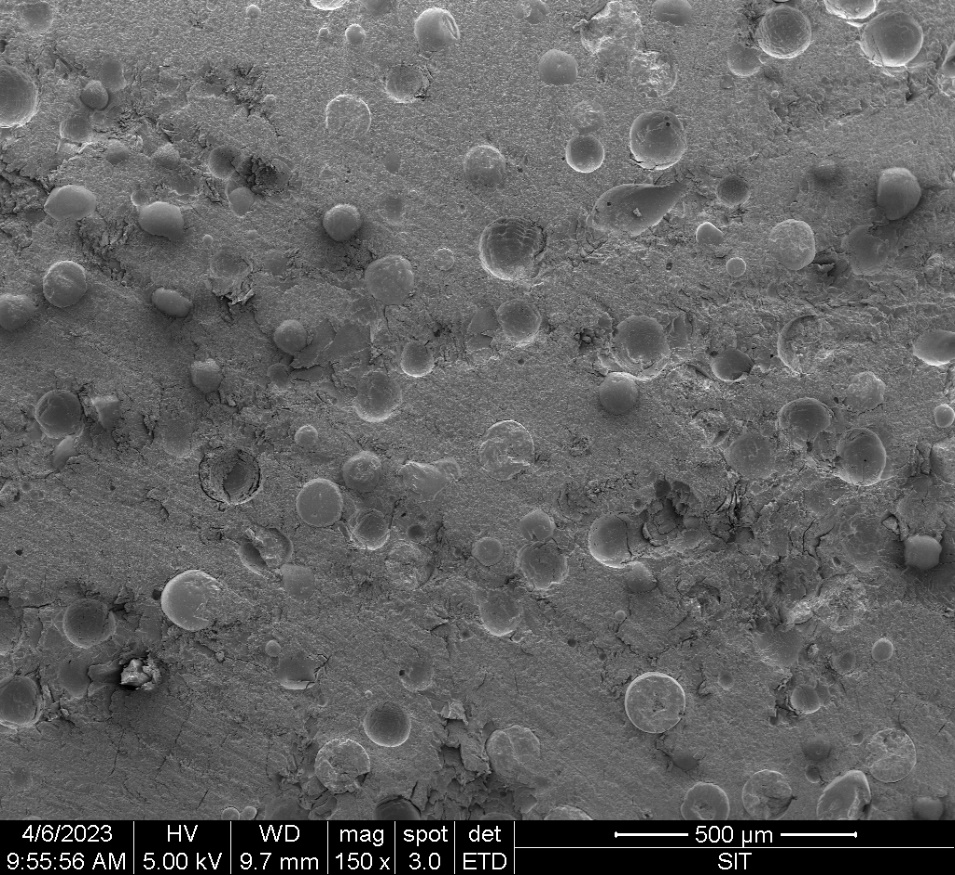

Supplement: S1 File — (DOCX) [file pone.0292878.s001.docx]
